# Supplementary material for: Randomized controlled trial parallel-group on optimizing community pharmacist’s care for the elderly: The influence of WhatsApp-Email delivered clinical case scenarios
Source: PLoS One. 2024 Oct 22;19(10):e0308448. doi: 10.1371/journal.pone.0308448 (PMC11495638; doi:10.1371/journal.pone.0308448)
Supplement: Attachment — Submitted filename: Rebuttable Letter.docx [file pone.0308448.s002.docx]

**Rebuttable Letter**

**Dear Editor,**

This is a rebuttal letter that responds to each point raised by the academic editor and reviewer.

1. The authors mention “DRPs are prevalent among older populations, with rates ranging from 34-56%”. Are these statistics reported from worldwide or only from UAE? Please clarify.

**Author Response:**

Globally the prevalence of DRPs among older people, ranging from 14.1% (USA) to 95.9% (the Netherlands). (32)

2. The rationale of the study intends to observe the impact of these educational interventions on the practices of the community pharmacists while dealing with elderly patients, however, the manuscript does not highlight any further of how this impact was determined?

**Author Response:**

As stated in the Outcome measure

The primary outcome measure was the improvement in the ability to identify DRPs. The differences in means between the interventions groups were taken as the outcome measure (the incidence of DRPs identified by pharmacists). The primary and secondary outcomes of the study were assessed using an electronic data collection sheet, delivered to pharmacists in all study groups and filled out over six months. The primary outcomes included the incidence, categories, and severity of DRPs, as well as the frequency and types of pharmacist interventions at both the physician and patient levels. The secondary outcomes included cost reduction per patient and time required to resolve a DRP.

3. Was there any practical observation of those community pharmacists in dealing with the elderly population after the dissemination of these educational interventions? Or was the identification of drug related problems assessed from the 24 clinical scenarios. Please justify.

The identification of drug related problems assessed from the 24 clinical scenarios

4. “The second intervention is the use of lengthy articles related to older patient care sent via email” How would the authors define “lengthy articles”

**Author Response:**

The research team conducted a search of the Cochrane Library for articles published after 2015 that documented DRPs, potentially inappropriate medications, and adverse drug reactions in elderly. After evaluating potential articles through three rounds, 24 articles were selected and sent to participants in PDF format.

5. The clinical scenarios are understandable in providing pharmacists with the necessary education and assessing their ability to identify DRPs, however, how this was accomplished through research articles is not documented enough. Please justify.

**Author Response:**

Traditional educational interventions, such as workshops and lectures, are often time-consuming, costly, and suffer from low engagement from learners.

In this study, we aimed to evaluate the effectiveness of two unique educational interventions on pharmacists' ability to optimize medication therapy plans in elderly. Specifically, we delivered clinical cases through WhatsApp and research articles via email to community pharmacists, and measured their interventions on elderly. As far as we know, this is the first study to investigate this type of educational intervention on pharmacists’ ability to identify DRPs in elderly.

6. **Methodology:**
   • What sampling method was employed to collect data from 127 pharmacies?

**Author Response:**

Randomly assigned to one of three groups using a 1:1:1 allocation ratio.

7. How the data for the population size of total community pharmacies in UAE were gathered? Based on this, how the minimum sample size for both community pharmacies and community pharmacists was determined?

**Author Response:**

The sample size was calculated using the equation: n = (Z^2 × p ×(1 - p)) / E^2 ^18^, where Z represents the Z-score corresponding to a 95% confidence level (1.96), p is the baseline incidence rate (0.0046), and E is the desired margin of error (0.00092). The calculation assumed a 20% improvement in pharmaceutical interventions for elderly medication therapy plans, including the detection of drug-related problems (DRPs). With these values, the sample size was estimated to be 141 per group, rounded up from 140.95. An additional 10% was added to account for an expected attrition rate of 10%, resulting in a final sample size of 156 pharmacists per group and a total sample size of 468 for the study.

8. The authors used different mediums (WhatsApp and email) for clinical case scenarios and research articles. Would this not affect the response received via email? How have the authors concluded that the low response for email sent articles was due to their length and not due to using email as a medium? Were these confounding factors taken into consideration?

**Author Response:**

Sending lengthy research articles through email did not result in a noteworthy increase in the number of DRPs identified by pharmacists. Furthermore, pharmacists who received clinical case scenarios through WhatsApp took less time to resolve a DRP and achieved a greater reduction in the cost compared to other groups. The use of this platform (WhatsApp) provided pharmacists with a unique educational experience, removing communication barriers with educators and potentially enhancing their engagement in educational programs.

9. “The cases, with a maximum word count of 150, were based on the panel members' experiences and previous literature”. No reference has been cited here to indicate the literature used to prepare 24 clinical case scenarios

**Author Response:**

Added

• How the reliability and validity of these clinical cases were ensured?

**Author Response:**

This available in the study procedure

(The cases were reviewed and validated by three independent assessors: a general practitioner, a community pharmacist, and a geriatrician.)

10. “The primary outcome measure was the improvement in the ability to identify DRPs”. Please consider mentioning the criteria for this improvement. Also was this improvement noted during the reporting of drug related problems from the clinical case scenarios or elderly patients coming to the community pharmacies after receiving these interventions? Please justify.

**Author Response:**

(The differences in means between the interventions groups were taken as the **outcome measure** (the incidence of DRPs identified by pharmacists). The primary and secondary outcomes of the study were assessed using an electronic data collection sheet, delivered to pharmacists in all study groups and filled out over six months. The primary outcomes included the incidence, categories, and severity of DRPs, as well as the frequency and types of pharmacist interventions at both the physician and patient levels. The secondary outcomes included cost reduction per patient and time required to resolve a DRP.)

11. For control, Group C, the authors mention no detail of how the outcome was measured from them. For instance, to compare with the study groups, it is assumed that they did not receive any clinical case scenarios and research articles. Therefore, how the competency of community pharmacists in this group was determined still entails comprehensive justification and explanation.

**Author Response:**

The differences in means between the interventions groups were taken as the **outcome measure** (the incidence of DRPs identified by pharmacists)

12. It is difficult to comprehend how the authors have determined the cost reduction per patient. Whether the cost reduction was determined by identifying the drug related problem in clinical case scenarios and then suggesting deprescribing of some medications leading to reduced medications and overall costs. If that is the case, how can we associate it with the impact of educational interventions afterwards? Please justify.

**Author Response:**

Reduction in cost required to resolve a DRP.

13. “The severity of DRPs was evaluated by a multidisciplinary expert panel, which met twice a month during the data reporting period and reviewed and rated each DRP reported by the participants” What was the rating criteria? Please clarify

**Author Response:**

DRPs rated according to the severity of the problems.

14. A total of 468 community pharmacists working in 131 pharmacies agreed to participate in the study and were assigned to three groups; the control group, Active Group A, and Active Group B. In what ratio, these pharmacists were allocated in these three groups? Please clarify. How selection and allocation biases were prevented in this case?

**Author Response:**

Randomly allocated to one of three arms using a 1:1:1 allocation ratio

15. Discussion:
    • The discussion section needs further improvement to present a detailed analysis of the findings. The findings should be compared with the results from other countries.
    • To update references on physicians’ acceptance of pharmacists’ recommendations and direct towards interprofessional collaboration, the following studies may be cited (This is optional and should only be taken as a suggestion for the improvement of the manuscript)
    1. DOI: 10.3389/fpubh.2024.1323102
    2. DOI: 10.2147/RMHP.S296113
    • The strengths, limitations and future prospects of the study must be mentioned comprehensively. More elaboration is required on how the authors have minimized different kinds of bias associated with RCTs such as selection bias, allocation bias, attrition bias, confounding bias etc. 
    References:
    • Several of the references need to be updated. Please check throughout.

**Author Response:**

In the discussion part, more citation and comparison were done, and references were added and updated. Limitations were elaborated.

**Corresponding Author**

**Ali Awadallah Saeed**
